# Overexpression of *CpADC* from Chinese Cherry (*Cerasus pseudocerasus* Lindl. ‘Manaohong’) Promotes the Ability of Response to Drought in *Arabidopsis thaliana*

**DOI:** 10.3390/ijms232314943

**Published:** 2022-11-29

**Authors:** Jiaxin Ran, Chunqiong Shang, Lina Mei, Shuang Li, Tian Tian, Guang Qiao

**Affiliations:** 1 Key Laboratory of Plant Resource Conservation and Germplasm Innovation in Mountainous Region (Ministry of Education), Institute of Agro-Bioengineering, College of Life Science, Guizhou University, Guiyang 550025, China; 2 Institute for Forest Resources & Environment of Guizhou, College of Forestry, Guizhou University, Guiyang 550025, China

**Keywords:** ‘Manaohong’ Cherry, polyamines, arginine decarboxylase, subcellular localization, drought stress

## Abstract

Polyamines (PA) play an important role in the growth, development and stress resistance of plants, and arginine decarboxylase (ADC) is one of the key enzymes in the biosynthetic pathway of polyamines. Previously, the transcriptional regulation of the ‘Manaohong’ cherry under the shelter covering was carried out, and the PA synthase-related genes, particularly the ADC gene, were differentially expressed as exposure to drought stress. However, the mechanisms of how ADC is involved in the response of cherry to abiotic stress (especially drought stress) are still unknown. In the present work, the full-length coding sequence of this gene was isolated and named *CpADC*. Bioinformatics analysis indicated that the coding sequence of *CpADC* was 2529 bp in length. Cluster analysis showed that *CpADC* had the highest homologies with those of sweet cherry (*Prunus avium*, XP_021806331) and peach (*Prunus persica*, XP_007200307). Subcellular localization detected that the *CpADC* was localized in the plant nucleus. The qPCR quantification showed that *CpADC* was differentially expressed in roots, stems, leaves, flower buds, flowers, and fruits at different periods. Drought stress treatments were applied to both wild-type (WT) and transgenic *Arabidopsi*s lines, and relevant physiological indicators were measured, and the results showed that the putrescine content of transgenic *Arabidopsis* was higher than that of WT under high-temperature treatment. The results showed that the MDA content of WT was consistently higher than that of transgenic plants and that the degree of stress in WT was more severe than in transgenic *Arabidopsis*, indicating that transgenic *CpADC* was able to enhance the stress resistance of the plants. Both the transgenic and WT plants had significantly higher levels of proline in their leaves after the stress treatment than before, but the WT plant had lower levels of proline than that of transgenic *Arabidopsi*s in both cases. This shows that the accumulation of proline in the transgenic plants was higher than that in the wild type under drought and high and low-temperature stress, suggesting that the transgenic plants are more stress tolerant than the WT. Taken together, our results reveal that, under drought stress, the increase in both expressions of *CpADC* gene and Put (putrescine) accumulation regulates the activity of ADC, the content of MDA and Pro to enhance the drought resistance of *Arabidopsis thaliana.*

## 1. Introduction

Polyamines (PAs), produced during the metabolism of organisms, is a kind of small molecular weight aliphatic nitrogenous alkali with biological activity [1]. Common polyamines mainly include putrescine (Put, diamine), spermidine (Spd, triamine), and spermine (Spm, tetraamine) in plants [2]. Polyamines have rich biological functions, playing important roles in plant growth and development, such as seed formation, flower bud differentiation, fruit ripening and abiotic stress [3,4,5]. As related studies have been reported, the main metabolic and regulatory pathways of polyamines have been largely revealed. Genes-encoded metabolic regulatory enzymes of polyamine biosynthesis in plants have been identified [6]. At the same time, some mutants related to polyamine biosynthesis have been obtained, and these transgenic plants and mutants have been studied, revealing that polyamines and anabolic-related enzymes were involved in physiological processes such as plant growth and development [7,8], and Arginine decarboxylase (ADC) was one of the key enzymes in the synthesis of plant polyamines [9]. Current studies have shown that ADC genes have been cloned in a large number of plants, such as mustard (*Brassica juncea*) [10], turnip (*Brassica rapa* var.) [11], apple (*Malus pumila*) [12], peach (*P. persica*) [7] and so on. Studies have shown that ADC plays an important role in participating in plant stress resistance [13]. For example, in the study of apple callus [14], although the Put content increased under salt stress, only the exogenous application of putrescine could alleviate the damage caused by salt stress. Some people think that endogenous putrescine has not reached the threshold for conversion to spermidine and spermidine [15]. Studies in which an oat *ADC* gene vector was constructed with an inducible promoter and introduced into rice plants showed that the transgenic plants obtained were significantly more resistant to salinity [16]. The low temperature, salt and dehydration treatments caught induced the transcriptional expression of the MdADC gene in apples, indicating that this gene was involved in the stress response of apples [12]. Some studies have reported that the expression of *ADC* was mostly related to the response of plants under stress conditions [17,18].

‘Manaohong’ cherry (*Cerasus pseudocerasus* Lindl.), a variant of sour cherry found in Nayong county, Guizhou province, China [19], approved and named by the Guizhou Provincial Variety Approval Committee, and was known as the “King of Southern China Cherry” [20]. Our previous study found that polyamine content in ‘Manaohong’ cherry leaves under rain culture was higher, and the expression of related enzyme genes, including ADC in polyamine synthesis pathway, was significantly increased [21,22]. The presence of rain shelter cultivation changes the growing environment of ‘Manaohong’ cherry plant to some extent, especially the water condition [23,24]. Studies have shown that the increased contents of Put, Spd or Spm are related to the improvement of stress tolerance in rice, Arabidopsis, tobacco and other plants [13,25,26]. Therefore, we speculate that *CpADC* plays an important role in the adaptation of the ‘Manaohong’ cherry to water deficit. Additionally, drought stress was among the most serious threats jeopardizing the economic yield of crop plants and fruits (For example, 50% deficit irrigation decreased both growth and pomological traits and the drought severity during the ripening phenological period has adverse effects on pomegranate (*Punica granatum* L.) fruit productivity, characteristics and composition.) [27,28]. As the severity of drought stress increased, with an accumulation of sodium and malondialdehyde, an incremental increase in osmolytes was observed, as well as an enhancement of the activity of antioxidant enzymes (peroxidase and catalase) [29]. To better understand the mechanisms of *CpADC* involved in drought stress and further clarify how the plant responds to abiotic stress is related to Put, Spd and Spm, we investigated the relationship between drought stress and *CpADC* gene function, and determined the contents of Put, Spd and Spm. *CpADC* was cloned and expressed in *Arabidopsis thaliana* to explore the mechanisms by which *CpADC* is involved in plant responses to drought stress.

## 2. Results

### 2.1. Obtaining of CpADC

**Figure 1:**
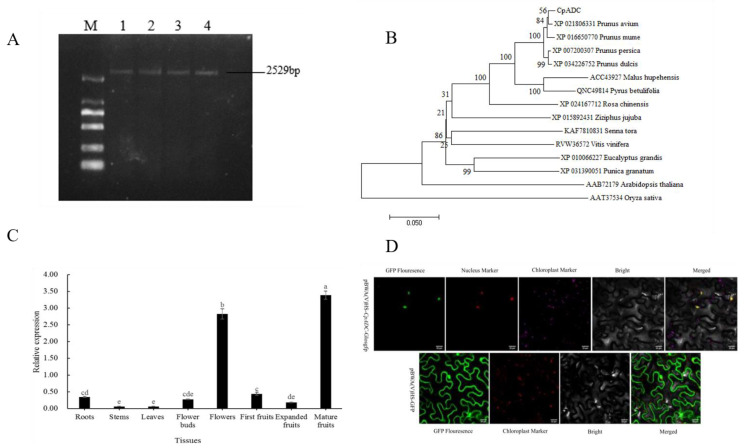
Obtaining of *CpADC* and its analysis. (**A**) Cloning of *CpADC* cDNA sequence from the leaves of cherry. Note: M: DL 2000 DNA marker; 1–4: *CpADC* Target fragment (**B**) Phylogenetic analysis of *CpADC*. (**C**) Tissue-specific expression of *CpADC* gene in cherry tissues by real-time quantitative PCR. Note: Different letters are used to indicate means that differ significantly (*p* ≤ 0.05); the same blow (**D**). Subcellular localization analysis of *CpADC*, which is localized in the nucleus in *N. benthamian* Tobacco leaves. Note: Respectively, from left to right: GFP fluorescence (green), nucleus fluorescence (red), merged images (green and red) and bright-field chloroplast images are shown. Scale bars = 10 μm.

The full-length coding sequence (CDS) of *CpADC* was isolated in the Chinese cherry leaves by designing primers (Table 1) in PCR amplification, and the length coding sequence of *CpADC* gene is 2529 bp (Figure 1A), including 22 open reading frames (ORFs). The longest ORF was 2178 bp and encodes the ADC zymogen with 735 amino acid residues, and the protein has a relative molecular mass of 77.7 KDa and an isoelectric point PI of 5.23.

### 2.2. Physiological Analysis and Sequence Alignment

Bioinformatics analysis revealed that it is an unstable protein with a typical PLPDE3 superfamily structure. Cluster analysis showed that the homologous relationship between *CpADC* and sweet cherry (XP_021806331) and peach (XP_007200307) with homology 98.90% and 98.07% was the highest, followed by flat peach and plum blossom with homology 98.90% and 98.07%, respectively. The amino acid sequences of 14 ADC enzymes from sweet cherry (*Prunus avium*, XP_021806331), plum blossom (*P. mume*, XP_016650770), honey peach (*P. persica*, XP_007200307), etc. were downloaded from the NCBI database, and the amino acids of the *CpADC* gene cloned from Chinese cherry were extracted. Sequences are compared, and a phylogenetic tree is constructed (Figure 1B). It can be seen in the phylogenetic tree that the relationship between *CpADC* and sweet cherry and honey peach has recently been clustered, which is consistent with the amino acid sequence similarity and multiple sequence alignments in conserved sequences.

### 2.3. Tissue-Specific Expression of *CpADC* Gene

The expression levels of *CpADC* gene in different tissues of ‘Manaohong’ cherry were analyzed using qRT-PCR, and the results showed that the *CpADC* is expressed in different tissues and varies depending on the tissue type. Furthermore, the highest expression was in mature fruits (55 days after full bloom), followed by flowers and the lowest expression in roots and leaves (Figure 1C).

### 2.4. *CpADC* Is Localized in the Nucleus

To further investigate the localization of the *CpADC* protein in plant cells, *CpADC* with deletion of termination codon was constructed to the expression vector pBWA(V)HS-GFP, which was named pBWA (V) HS-*CpADC*-GFP is driven by 35S promoter in this study. The control (GFP) and pBWA (V) HS-*CpADC*-GFP fusion protein expression vector were transformed into *N. benthamian* leaves by agrobacterium-mediated method while monitored by a Confocal microscope.

Confocal images showed that GFP fluorescence was green, nuclear marker fluorescence was red, fusion fluorescence was yellow, the colocalized marker was red, and the chloroplast fluorescence signal was also red, later adjusted to rose red for easy identification (Figure 1D). The results show that *CpADC* is localized in the nucleus, in agreement with the results predicted by the online software Plant-mPloc server.

### 2.5. Transformation and Characterization of Transgenic Arabidopsis thaliana

**Figure 2:**
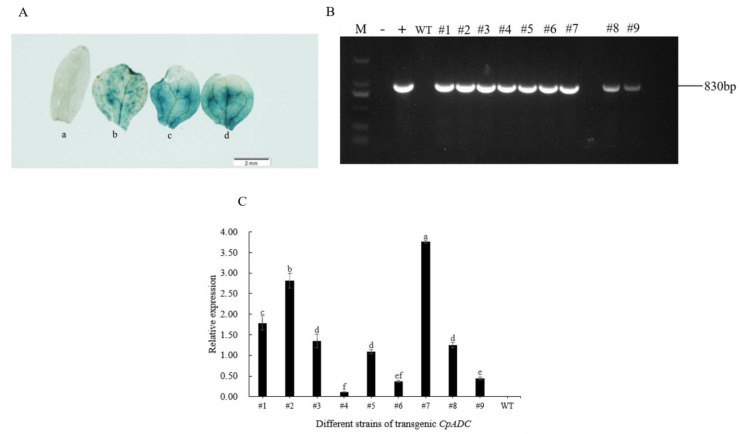
Identification and expression analysis of transgenic strains (**A**) The histochemical staining of GUS from the transgenic plants. Note: a: WT; b,c,d: transgenic Arabidopsis plant. Scale bars = 2 mm (**B**) DNA detection of T1 generation of transgenic Arabidopsis plant. Note: M: DL 2000 DNA Marker; -: H_2_O, +: recombinant vector plasmid, #1–#9: transgenic Arabidopsis plant lines. (**C**) Expression analysis of *CpADC* in nice transgenic lines by qRT-PCR. #1–#9: transgenic Arabidopsis plant lines; Note: Different lowercase letters are used to indicate means that differ significantly (*p* ≤ 0.05), WT: Colombia wild type Arabidopsis.

As shown that the plant overexpression vector was constructed, using *Arabidopsis thaliana* (Colombia wild type) as material, *CpADC* was genetically transformed into *Arabidopsis thaliana* by floral dip by *Agrobacterium*-mediated transformation, identification of plant tissues by GUS staining (Figure 2A), PCR method, and multiple generations of resistance screening, nine homozygous strains with overexpression of T3 generation were obtained (Figure 2B). To further confirm the *CpADC* transcript levels of positive overexpression lines, real-time PCR was applied to four randomly selected transgenic lines (#1–#9) and WT plants under normal conditions (Figure 2C) with the actin7 (Table 1, At-actin7-F/ At-actin7-R) was selected as a reference gene in *Arabidopsis thaliana*. The transgenic lines #7 with the highest *CpADC* transcript level and the lowest transgenic lines #4 were selected for further analysis along with the corresponding untransformed WT.

### 2.6. Quantification of Free Polyamine Contene

**Figure 3:**
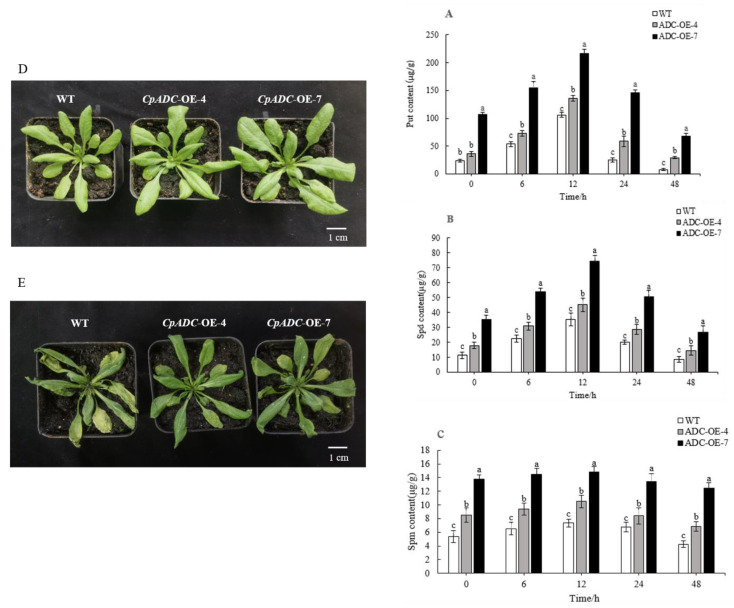
PAs content in 30 d leves of WT, CpADC-OE-4 and CpADC-OE-7. (**A**) Free putrescine content. (**B**) Free spermidine content. (**C**) Free spermine content. Values are means of three biological replicates. Note: Different lowercase letters are used to indicate means that differ significantly (*p* ≤ 0.05); (**D**,**E**) Arabidopsis lines before and after drought treatment, respectively.

High-performance liquid chromatography (HPLC) was used to determine the contents of endogenous polyamines in transgenic *Arabidopsis* and WT under simulated drought stress in a PEG solution. Figure 3A–C shows a trend of increasing and then decreasing polyamine content in both transgenic and WT plants, with a more pronounced trend for Put and Spd, while the change in Spm content is not significant. All peaked at 12 h; compared to the wild type, the Put content of the transgenic strain *CpADC*-OE-7 and strain *CpADC*-OE-4 increased approximately 2.05-fold and 1.28-fold, respectively (Figure 3A), and the Spd content of the transgenic strain *CpADC*-OE-7 and strain *CpADC*-OE-4 increased approximately 2.11-fold, and 1.65-fold, respectively (Figure 3B) and the transgenic strain *CpADC*-OE-7 and strain *CpADC*-OE-4 showed an increase in Spm content by about 2.01-fold and 1.42-fold, respectively (Figure 3C). Phenotypically, the leaves of transgenic Arabidopsis thaliana appeared significantly different from those of the WT, which the leaves of the transgenic *Arabidopsis thaliana* were significantly larger than those of the WT (Figure 3D) and under drought treatment, *Arabidopsis thaliana* wilted and yellowed, but the transgenic strains did not wilt as severely as the wild type, and the physiological indicators corresponded (Figure 3E). Thus, overexpression of the *CpADC* gene in Chinese cherry increased the endogenous polyamines (Put, Spd and Spm) in Arabidopsis, and overexpression of the *CpADC* gene led to an increase in polyamine (PAs) content that played a role in the growth and development of *Arabidopsis*.

### 2.7. Exposure to Drought Environment Led to Enhanced ADC Activity in AtADC Overexpression Line

**Figure 4:**
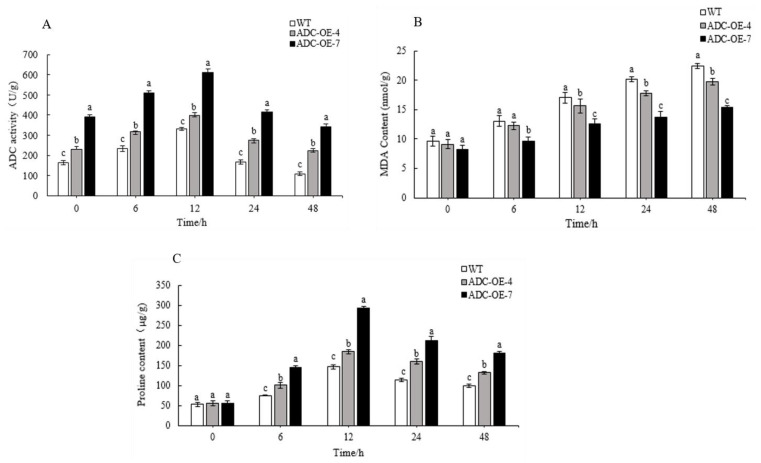
Determination of physiological parameters of transgenic lines and WT. (**A**) ADC activity in WT, *CpADC*-OE-4 and *CpADC*-OE-7. (**B**) MDA contents in WT, *CpADC*-OE-4 and *CpADC*-OE-7. (**C**) Proline content in WT, *CpADC*-OE-4 and *CpADC*-OE-7. Note: Different lowercase letters are used to indicate means that differ significantly (*p* ≤ 0.05).

The ADC activities of the transgenic *Arabidopsis* and WT under simulated drought stress, stress was measured using ELISA (Figure 4A). The results showed that ADC activities of both transgenic and WT leaves showed a trend of increasing and then decreasing, and both reached the maximum at 12 h stress treatment. The ADC activity of *CpADC*-OE-7 reached the highest, which was 1.84 times that of the WT and 1.5 times that of the *CpADC*-OE-4. This result suggests that exposure to a drought environment leads to enhanced ADC activity in *CpADC* overexpression lines.

### 2.8. MDA and Pro Levels Were Increased in Transgenic Arabidopsis Plant under Drought Tress

In particular, the greater the MDA content, the more the plant is exposed to adversity and the less it is resistant to stress; Conversely, the resistance to stress is high. Hence, we detected the variations of MDA content in transgenic *Arabidopsis* strains *CpADC*-OE-7, *CpADC*-OE-4 and WT under drought stress, and the results showed that there was an increasing trend in both transgenic and WT (Figure 4B). In contrast, the increase in MDA content was more pronounced in WT than in the transgenic strain. These results indicate that under drought stress, the cell membrane system of WT is disrupted to a greater extent than that of the transgenic lines. Therefore, the transgenic lines show resistance to drought stress. Proline accumulation and metabolism are associated with mechanisms of abiotic stress avoidance in plants, which generally improve osmotic stress tolerance. In the research, the free proline content of each strain was measured under drought stress, and the result showed that there was no significant difference in all lines under normal culture (0 h) while the accumulates significantly highest proline by 12 h. Moreover, *CpADC*-OE-7 and *CpADC*-OE-4 had significantly higher proline content than WT (Figure 4C). These results suggested that under drought stress, transgenic strains were more drought tolerant than WT. Thus, these results support that *CpADC* gene played a role in drought stress response.

## 3. Discussion

With the reports of related studies, the main metabolic and regulatory pathways of polyamines have been basically revealed. Genes-encoded metabolic regulatory enzymes of polyamine biosynthesis in plants have been identified [30]. Its biosynthetic metabolic pathway has received much attention [31], and spermidine decarboxylase (ADC) is one of the key enzymes in the synthesis of plant polyamines, and putrescine (Put) is produced by spermidine decarboxylase catalyzing the synthesis of herring spermine followed by conversion [7,32]. Based on the current research progress, the physiological mechanism of PAs to improve plant drought resistance may be based on the following aspects: PAs can eliminate the active oxygen free radicals, reduce the degree of membrane lipid peroxidation, and protect the plasma membrane and biological macromolecules from damage by affecting the activities of nucleases and proteases, especially the protective enzymes; Secondly, PAs can also be used as an osmotic regulator to regulate the content of osmotic regulating substances, reduce the osmotic potential and enhance the water absorption [33]. However, thus far, little information is available on the ADC gene in cherries. In this study, we have cloned the coding sequences of the *CpADC* gene in ‘Manaohong’ cherry, which was the first cloned PAs-related synthase gene in this species. Phylogenetic analysis showed that the *CpADC* had more homologous to ADC from sweet cherries and peaches, partially reflecting the close genetic relationship between sweet cherries and peaches, suggesting that the gene is highly conserved among fruit plants. The highest expression of *CpAD*C was found in ripe fruit and flowers, followed by flower buds and roots, while the lowest expression was found in leaves and stems. Tissue-specific analysis suggests that this gene may be widely involved in the growth and development of the ‘Manaohong’ cherry and may have an important role in fruit ripening development.

The ADC carried out the production of agmatine from arginine, which was the precursor of the first PA known as putrescine; Subsequently, putrescine was turned into the higher PAs, spermidine and spermine [34]. Previous studies had proposed that the different functions of PAs in plants were associated with differential activation of the Put biosynthetic pathway catalyzed by ADC [35]. The study suggested that the same enzyme (ADC) could play different roles depending on its subcellular localization, e.g., the chloroplastic ADC form could be involved in photosynthesis, whereas the nuclear ADC form could play a role in cellular signalling [36]. In *Arabidopsis thaliana,* PA production occurred only from arginine, and this step was initiated by two ADC paralogues, *AtADC1* and *AtADC2* [37]. Similarly, subcellular localization resulted in this research showed that the fusion expression vector GFP fluorescent protein material was present in the nucleus, confirming that the metabolites of the *CpADC* gene were expressed in the nucleus, i. e., that the ADC protein acts mainly in the nucleus, which validated that the *CpADC* gene functions mainly by the ADC pathway in transgenic Arabidopsis. Furthermore, ADC localization in the nucleus somehow suggested that *CpADC* might function as a regulator for gene expression. The same implication was also documented in mustard [10].

Pas, as a class of very potent bioactive substances, were closely related to plant physiological activities, such as participation in plant growth and development and physiological stress responses [38]. Current research on PAs focuses on Put, Spd and Spm, and the levels of different endogenous PAs and the conversion between them had positive physiological functions in plant stress responses. Increased levels of endogenous Put, Spd and Spm were an adaptive response of plants to adversity, and the conversion ability of Put to Spd and Spm became an indicator of plant tolerance to multiple stresses [5]. Previous study using oat ADC transgenic genetic transformation of rice found that overexpression of this gene increased plant drought resistance under drought conditions [15]. Overexpression of *AtADC2* and increased Put content also improved drought resistance in Arabidopsis by inducing stomatal closure to reduce water loss [13], and citrus Overexpression of PtADC promoted Put synthesis in Arabidopsis adc2 mutants [39]. Additionally, there was a study treated wheat seedlings with 0.5 MPa polyethylene glycol (PEG) 6000 for 7 d and found a significant increase in free Spd and Spm content in drought-tolerant wheat leaves, while only Put content increased in sensitive wheat leaves [40]). The overexpressed and genetically transformed the *PtADC* gene in citrus, which improved the drought and cold resistance of the transgenic plants in addition to the dwarfism exhibited by the transgenic strains [41], and overexpression of the *MdADC* gene in apple improved the resistance to biotic stresses such as drought, low-temperature tolerance and salt resistance in tobacco when using drought, low temperature and salt stress treatments [42].

Similarly, in the present study, *Arabidopsis thaliana* was genetically modified using overexpression of the *CpADC* gene and showed higher levels of putrescine than the WT under drought stress. Measurement of ADC activity by ELISA different treatments revealed that the transgenic *Arabidopsis* had higher ADC activity than that of the WT and showed a positive correlation with the putrescine accumulation. Thus, these results showed that under drought stress (PEG simulation), the transgenic plants accumulated putrescine rapidly within a short period of time, and the ADC activity was enhanced to participate in the plant’s stress resistance response.

MDA is the final reaction product of membrane lipid peroxidation in plants. The amount of MDA content was usually used as an indicator of membrane lipid peroxidation, which can reflect the degree of stress tolerance in plants [43]. MDA variation analysis showed that the increase in MDA content in the transgenic strains was not as pronounced as in the wild type (Figure 4B), and the cell membrane system was less disrupted in the strains with high expression of the transgene than in those with low expression, which promoted the drought tolerance of Arabidopsis thaliana. Furthermore, in plants, Pro is one of the high protein fractions of plants. Its accumulation changes significantly when subjected to adversity stress, and the accumulation of proline is closely related to plant stress resistance [44]. Pro variation analysis showed a significant increase in Pro content in the transgenic Arabidopsis compared to the WT and that the *CpADC* gene plays a role in drought stress responses.

In conclusion, we have cloned the coding sequence of the ‘Manaohong’ cherry *CpADC* gene, the first cloned ADC in this species. Our results reveal that, under drought stress, increased expression of *CpADC* gene and Put accumulation regulates the activity of ADC, MDA and Pro content to enhance the drought resistance in *Arabidopsis thaliana*.

## 4. Materials and Methods

### 4.1. Plant Materials Growth Conditions and Drought Stress Treatment

Materials used in the experiment were 5-year-old cherry plants in a public orchard in Nayong (26°77′ N; 105°38′ E), which is in the Northwest of Guizhou province, China. The different tissues: roots, stems, leaves, flower buds, flowers, first fruits (35 days after full bloom), expanded fruits (45 days after full bloom) and mature fruits (55 days after full bloom) were collected for the gene clone and expression respectively of the cherry *ADC* gene from January to April in 2021.

The plants of wild-type (WT) *Arabidopsis thaliana* and *CpADC*-OE transgenic lines were grown in an artificial climate chamber for 30 d under conditions of 23 °C, with 16 h of light/8 h of darkness and 65–75% relative humidity. Plants with uniform and healthy growth were selected for drought stress treatment, and physiological parameters were measured. Drought stress treatment: After watering the plant with the same amount of water (maintaining the same water potential, all plant lines were planted with the same soil weight and area), we used 20% polyethylene glycol to simulate the drought treatment: The leaves were collected at 0 h, 6 h, 12 h, 24 h and 48 h stress treatments, flash-frozen in liquid nitrogen and stored in an ultra-low temperature refrigerator.

### 4.2. Cloning of *CpADC* Cording Sequence

Total RNA in the leaves was extracted by polysaccharide polyphenol plant kit (SENO, Zhangjiakou, China) according to the manufacturer’s instructions, and the first strand of cDNA was synthesized using a PrimeScript RT reagent Kit with gDNA Eraser (TaKaRa, Beijing, China) according to the manufacturer’s protocol. Based on the ADC EST sequences from the cherry transcriptome data, the upstream and downstream primers *CpADC*-F1/ *CpADC*-R1 (Table 1) were designed respectively by Primer 5.0 software. They were synthesized by Sangon Biotech Company (Shanghai, China). The total reaction volume of RT–PCRs was 10 μL: 5 μL of PreMix (Tiangen, Wuhan, China), 2 μL of template cDNA, 2 μL of ddH_2_O, and 5 μL of reverse and forward primers (10 μmol L^−1^). The PCR program included an initial denaturation step at 95 °C for 10 s, followed by 35 cycles of 5 s at 95 °C, and annealing and extension at 58 °C for 30 s. The PCR product was detected on a 1% agarose gel with GoldView (10,000×) (Solarbio, Beijing, China) staining, imaging under the SYNGENE G: Box system (Syngene, Shanghai, China) and was recovered using Takara MiniBest Agarose Gel DNA Extraction Kit (9762) gel recovery kit. The recovered product was directly connected to the pEASY-Blunt cloning vector, and the total volume of the ligation system was 5 μL containing 1.0 μL pEASY-Blunt cloning Vector and 4.0 μL PCR product. After transfection for 15–25 min at 37 °C, Trans1-T1 competent cells were transformed and plated on ampicillin-containing plates for 16–20 h at 37 °C. After incubation with LB liquid, the clones were identified by PCR as positive clones and then sequenced by Industrial Bioengineering Co., Ltd. (Shanghai, China).

### 4.3. Sequence Analysis of CpADC

The amino acid sequence of the *ADC* gene was deduced and compared using NCBI BLAST server (http://www.banana-genome.cirad.fr/blast, accessed on 10 April 2021), and the nucleic acid and amino acid sequence of the enzyme gene was uploaded to GenBank by Sequin. The molecular weights (kDa) and isoelectric points (pI) of the *CpADC* proteins were predicted using the ProParam tool (http://www.expasy.ch/tools/pitool.html, accessed on 10 April 2021). Multiple sequence alignments and Cluster analysis were performed using the Clustal W software (MEGA 7.0), combined with *ADC* gene sequences from other plants in GenBank. The phylogenetic tree was constructed using MEGA 6.0 software by the Neighbor-joining tree method.

### 4.4. Subcellular Localization of *CpADC* Protein

Primer ADC-F2, and ADC-R2 (Table 1) were designed with the first strand of cherry cDNA as a template, and the amplified target fragment was recovered by gel. After detection, the termination code was removed and ligated into the expression vector pBWA (V) HS-GLosgfp, 10 μL of E. coli competent DH5α was cultured overnight, the colony PCR was performed, sequenced and the plasmids were extracted for later use. Agrobacterium competent cell GV3101 was taken out, placed on ice for plasmid transformation, added liquid LB medium 600 μL, and cultured with shaking for 3 h (temperature 28 °C, shaker speed 150 r·min^−1^); The cells were collected by centrifugation at room temperature, resuspended the bacterial liquid, and evenly spread on a solid LB plate containing 50 mg·L^−1^ kanamycin (Kan) and 100 mg·L^−1^ rifampicin (Rif), and placed incubate at 28 °C for 48 h. The monoclonal strains were picked up in 1 mL LB liquid medium and cultured with shaking at 28–30 °C for 24 h. Transfer 1 mL of Agrobacterium cultured overnight to 25 mL LB liquid medium with 50 mg·L^−1^ Kan and 100 mg·L^−1^ Rif, and then add 2 µL of 100 mmol·L^−1^ acetyl Syringone (AS) and 100 µL 0.5 mol·L^−1^ MES were cultured in a shaker at 28 °C. The cells were collected by centrifugation at room temperature. Resuspend the bacteria in LB medium containing 10 mmol·L^−1^ MgCl_2_, add 2 μL 100 mmol·L^−1^ AS per ml of bacterial solution, and let stand for more than 3 h. For the tobacco leaves that were taken in the growing period, pierce some small holes on the reverse side of the leaves with a needle, put the infection solution into a 5 mL syringe, pressed the reverse side of the syringe with your thumb to inject the liquid from the lower surface of the leaves into the tobacco leaves. Cultured in the dark for 72 h, the leaf epidermis was removed, and the fluorescence signal was detected by a laser confocal fluorescence microscope (EVOS700, China). The parameters are as follows: chlorophyll fluorescence excitation light at 560 nm, and emission light at 650 nm; Green fluorescent protein (GFP) excites light at 488 nm, and emits light at 510 nm; The nuclear marker fusion fluorescent protein excites light at 561 nm, and emits light at 580 nm.

### 4.5. Analysis of *CpADC* Gene Expression by Quantitative Real-Time PCR (qRT-PCR)

Based on *CpADC* gene sequence, the primer pairs were designed for qRT-PCR listed in Table 1 (ADC-qF/ADC-qR). The PCR reaction consisted of 5 µL of 2 × SYBR Green PCR Master Mix (TaKaRa), 0.5 μL primers, 1 μL cDNA template of different tissues which contained roots, stems, leaves, flower buds, flowers, first fruits (35 days after full bloom), expanded fruits (45 days after full bloom) and mature fruits (55 days after full bloom), and 3 μL ddH_2_O up to the total volume of 10 µL. The qRT-PCR amplification was performed by an initial denaturation step at 95 °C for 30 s, followed by 40 cycles of 5 s at 95 °C, and annealing and extension at 60 °C for 30 s. The EF gene (Table 1) was selected as a reference gene as per the previous report [45]. Transcript abundance was normalized against the reference gene and the relative gene expression was calculated using the 2^−ΔΔCt^ method using the CFX Connect™ Real-Time PCR Detection System (Bio-Rad Laboratories, Hercules, CA, USA).

### 4.6. Generation of Transgenic Plants Arabidopsis thaliana by Agrobacterium Tumefaciens Mediated Transformation

*CpADC* cDNA was amplified with specific primers (*CpADC*-cF/*CpADC*-cR) containing BsaI or BsmBI restriction sites. The *CpADC* target fragment was constructed on the expression vector pBWA(V)KS-GUS that 35 s promoter driven by the seamless cloning method (Table 1), and the successful construction of the plant expression vector pBWA(V)KS-*ADC*-GUS was verified by the PCR product. The newly constructed pBWA(V)KS-*ADC*-GUS plant expression vector was transferred into A. tumefaciens strain GV1301 by heat shock. Later, the overexpression vector was used for the transformation of *Arabidopsis thaliana* by floral dip by Agrobacterium-mediated transformation [46]. The *Arabidopsis thaliana* seeds received after infection were vernalized for 2–3 days at 4 °C, and sterilized with 75% ethanol and 10% sodium hypochlorite for 30 s and 8–10 min in a sterile environment, respectively. After washing with aseptic water 3–5 times, evenly the sterilized seeds were sprinkled on the medium (MS + 50 mg/L Timentin + 50 mg/L Kanamycin) containing resistant hormones and cultivated for 10–14 days in an artificial climate box at 23 °C under 16 h light/8 h dark conditions. GUS identification of the grow-up transgenic *Arabidopsis* leaves was performed using the Plant Tissue GUS Staining Kit, following the kit method, as the Arabidopsis seedlings grew to about 30 d. The *Arabidopsis* genomic DNA identified by GUS was then extracted using the new Plant Genomic DNA Extraction Kit (Tiangen, Wuhan, China) according to the instructions, and specific primers (Table 1, *CpADC*-JF/*CpADC*-JR) were designed for positive PCR identification. The PCR reaction system and procedure are the same as described above for the *CpADC* gene cloning.

### 4.7. Quantification of Free Polyamine Content

Free PAs were analyzed by high-performance liquid chromatography (Waters e2695, Guizhou, China) separation of benzoyl chloride-derivatized PAs. The material handling and derivatization reactions were referenced by Zhang, et al. (2020) [32]. Grind 0.2 g plant leaves with liquid nitrogen, add the ground powder to the centrifuge tube of 2 mL, add precooled 5% perchloric acid 1.5 mL, vortex mix, and put it in a refrigerator at 4 °C or ice for 1 h. 30 min was centrifuged by 5000× *g* at 4 °C, and the supernatant of 1 mL was prepared (stored at 4 °C). Then the supernatant of 1 mL was added to the 10 mL centrifuge tube, 10 µL benzoyl chloride was added to it, and then 1 mL 2 mol/L NaOH solution was added for 20 s. After bathing at 37 °C for 30 min, adding 2 mL saturated NaCl solution, mixing and adding 2 mL ether to extract, and shaking violently. 6000× *g* centrifugation for 5 min, 1 mL ether phase was placed in a 2 mL centrifuge tube, dried with nitrogen, then resuspended with 1 mL ether, and dried again with nitrogen. After adding 500 µL 60% methanol vortex to dissolve, the 0.22 µm filter membrane was filtered into the inner lining tube and placed in the brown injection bottle to be tested.

### 4.8. Arginine Decarboxylase Activity Determination, MDA and Pro Content Measurement

The activity of ADC is determined by enzyme-linked immunosorbent assay (ELISA). The pre-grinded sample was weighed and added to 0.1 mM, pH7.4 phosphate buffer pre-cooled to 4 °C (Phosphate buffered saline, PBS), homogenized according to the weight and volume of 1:9. It was then added to the glass homogenizer for manual grinding, extracted for 2 h at 4 °C, centrifuge at 3000 r for 5 min to take the supernatant for use. Then we followed the instructions that came with the kit.

For the determination of malondialdehyde (MDA) and proline (Pro) content, we followed the relevant kit (Suzhou Comin Biotechnology Co., Ltd., Suzhou, China) instructions for the experiment. Additionally, for all the leaves used for biochemical analysis, a fourth leaf from the transgenic Arabidopsis plant was taken, and biological replicates were performed.

### 4.9. Statistical Analysis

All data reported in the experiment were assessed using Duncan and Tukey test at significantly different levels with the SPSS 21.0 statistics package (Chicago, IL, USA). All the presented data are the means and standard deviations (SDs) of at least three replicates. The graphs were constructed with Excel and Origin 9.0 (Origin Lab, Northampton, MA, USA).

## 5. Conclusions

In conclusion, the coding sequence of the ‘Manaohong’ cherry *CpADC* gene has been cloned, the first cloned ADC in this species. Phylogenetic analysis showed that the *CpADC* had more homologous to ADC from sweet cherries and peaches, partially reflecting the close genetic relationship between sweet cherries and peaches, suggesting that the gene is highly conserved among fruit plants. The highest expression of *CpAD*C was found in ripe fruit and flowers, followed by flower buds and roots, while the lowest expression was found in leaves and stems. Tissue-specific analysis suggests that this gene may be widely involved in the growth and development of the ‘Manaohong’ cherry and may have an important role in fruit ripening development. subcellular localization resulted in this research showed that the fusion expression vector GFP fluorescent protein material was present in the nucleus, confirming that the metabolites of the *CpADC* gene were expressed in the nucleus, i.e., that the ADC protein acts mainly in the nucleus, which validated that the *CpADC* gene functions mainly by the ADC pathway in transgenic Arabidopsis. Measurement of ADC activity by ELISA different treatments revealed that the transgenic Arabidopsis had higher ADC activity than that of the WT and showed a positive correlation with the putrescine accumulation. Thus, these results showed that under drought stress (PEG simulation), the transgenic plants were able to accumulate putrescine rapidly within a short period of time, and the ADC activity was enhanced to participate in the plant’s stress resistance response. MDA variation analysis showed that the increase in MDA content in the transgenic strains was not as pronounced as in the wild type. Pro variation analysis showed a significant increase in Pro content in the transgenic Arabidopsis compared to the WT and that the *CpADC* gene plays a role in drought stress responses. Thus, these results reveal that, under drought stress, increased expression of *CpADC* gene and Put accumulation regulates the activity of ADC, MDA and Pro content to enhance the drought resistance in *Arabidopsis thaliana*.

## Figures and Tables

**Table 1:** The sequences of primers used for *CpAD*C cloning, its expression quantification and so on.

Primer Name	Primer Sequence	Purpose
*CpADC*-F1	GCCAGCCATTACAAACTCACAA	Clone for CpADC
*CpADC*-R1	TAGAAGAGGCGGGAATAGGG	
*CpADC*-F2	CAGTCGTCTCACAACATGCCGGCCCTGGCTTGTTG	Subcellular localization of *CpADC* protein
*CpADC*-R2	CAGTCGTCTCATACAAGCACAGCAGTAAGACCACT	
*CpADC*-qF	AGACGTTCCCAATAGTCCCGA	qRT-PCR for CpADC
*CpADC*-qR	TGATAAGCCCCGCCCAAG	
*CpADC*-cF	CAGTCGTCTCACAACATGCCGGCCCTGGCTTGTTG	Constructing plant expression vector
*CpADC*-cR	CAGTCGTCTCATACATCAAGCACAGCAGTAAGACC	
*EF*-F	TGAGAGGCTGACTGTGCTGTTC	Internal reference gene for qRT-PCR
*EF*-R	GGAGTAGTGGCATCCATCTTGTT	
*CpADC*-JF	CCCACCCACGAGGAGCAT	Characterization of transgenic Agrobacterium tumefaciens
*CpADC*-JR	GCGAGCAAACAGAGCCAGAG	
At-action7-F	AGCTAGAGACAGCCAAGAGC	Internal reference gene for transgenic line qRT-PCR
At-action7-R	GCTTCCATTCCGATGAGCGA	

## Data Availability

Not applicable.
